# Pathogenesis of Velogenic Genotype VII.1.1 Newcastle Disease Virus Isolated from Chicken in Egypt via Different Inoculation Routes: Molecular, Histopathological, and Immunohistochemical Study

**DOI:** 10.3390/ani11123567

**Published:** 2021-12-15

**Authors:** Yassmin EL-Morshidy, Walied Abdo, Ehab Kotb Elmahallawy, Ghada Allam Abd EL-Dayem, Ahmed El-Sawak, Nagwan El-Habashi, Samah M. Mosad, Maha S. Lokman, Ashraf Albrakati, Samah Abou Asa

**Affiliations:** 1 Department of Veterinary Pathology, Agriculture Research Center (ARC), Animal Health Research Institute (AHRI), P.O. Box 246, Dokki, Giza 12618, Egypt; Dr.jessy.mail@gmail.com; 2 Department of Veterinary Pathology, Faculty of Veterinary Medicine, Kafrelsheikh University, Kafr El Sheikh 33516, Egypt; waliedsobhy40@gmail.com (W.A.); elsawak1953@yahoo.com (A.E.S.); nagwan_hab@yahoo.com (N.E.-H.); ssabouasa@yahoo.com (S.A.A.); 3 Department of Zoonoses, Faculty of Veterinary Medicine, Sohag University, Sohag 82524, Egypt; 4 Department of Poultry Diseases, Agriculture Research Center (ARC), Animal Health Research Institute (AHRI), P.O. Box 246, Dokki, Giza 12618, Egypt; anasesra@gmail.com; 5 Department of Virology, Faculty of Veterinary Medicine, Mansoura University, Mansoura 35516, Egypt; dr.sama786@yahoo.com; 6 Biology Department, College of Science and Humanities, Prince Sattam bin Abdul Aziz University, Alkharj 11942, Saudi Arabia; ms.hussein@psau.edu.sa; 7 Department of Zoology and Entomology, Faculty of Science, Helwan University, Cairo 11795, Egypt; 8 Department of Human Anatomy, College of Medicine, Taif University, P.O. Box 11099, Taif 21944, Saudi Arabia; a.albrakati@tu.edu.sa

**Keywords:** histopathological, immunohistochemical, molecular, NDV-genotype VII.1.1, chickens, different routes

## Abstract

**Simple Summary:**

Newcastle disease (ND) is a worldwide distributed highly infectious disease of wild and domestic birds. Regardless of vaccination procedures, little information is available about the pathogenicity and genetic characteristics of the circulating virus in the Egyptian environment. The current study was undertaken to estimate the role of the different inoculation routes in the pathogenicity of NDV. Therefore, induction of the infection was done with *NDV-CH-EGYPT-F42-DAKAHLIA-2019* (VNDV) isolated strain in 28 day old white Ross male broiler chicks via intraocular, choanal, intranasal, mixed oculo-nasal routes. Interestingly, a series of findings including mortality rates, severity of clinical and histopathological lesions, and intensity of viral nucleoprotein immunolabeling were reported to be different according to the routes of inoculation. Clearly, the present findings provide novel descriptive and comparative histopathological and immunohistochemical findings about this virus in Egypt, and the obtained data may be useful for different vaccination strategies against NDV.

**Abstract:**

Newcastle disease virus (NDV) remains a constant threat to the poultry industry. There is scarce information concerning the pathogenicity and genetic characteristics of the circulating velogenic Newcastle disease virus (NDV) in Egypt. In the present work, NDV was screened from tracheal swabs collected from several broiler chicken farms (*N* = 12) in Dakahlia Governorate, Egypt. Real-time reverse transcriptase polymerase chain reaction (RRT-PCR) was used for screening of velogenic and mesogenic NDV strains through targeting F gene fragment amplification, followed by sequencing of the resulting PCR products. The identified strain, namely, NDV-CH-EGYPT-F42-DAKAHLIA-2019, was isolated and titrated in the allantoic cavity of 10 day old specific pathogen-free (SPF) embryonated chicken eggs (ECEs), and then their virulence was determined by mean death time (MDT) and intracerebral pathogenicity index (ICPI). The pathogenicity of the identified velogenic NDV strain was also assessed in 28 day old chickens using different inoculation routes as follows: intraocular, choanal slit, intranasal routes, and a combination of both intranasal and intraocular routes. In addition, sera were collected 5 and 10 days post inoculation (pi) for the detection of NDV antibodies by hemagglutination inhibition test (HI), and tissue samples from different organs were collected for histopathological and immunohistochemical examination. A series of different clinical signs and postmortem lesions were recorded with the various routes. Interestingly, histopathology and immunohistochemistry for NDV nucleoprotein displayed widespread systemic distribution. The intensity of viral nucleoprotein immunolabeling was detected within different cells including the epithelial and endothelium lining, as well as macrophages. The onset, distribution, and severity of the observed lesions were remarkably different between various inoculation routes. Collectively, a time-course comparative pathogenesis study of NDV infection demonstrated the role of different routes in the pathogenicity of NDV. The intranasal challenge was associated with a prominent increase in NDV lesions, whereas the choanal slit route was the route least accompanied by severe NDV pathological findings. Clearly, the present findings might be helpful for implementation of proper vaccination strategies against NDV.

## 1. Introduction

Newcastle disease (ND) is a highly contagious viral disease which infects a wide variety of domestic and wild bird species [1,2,3]. The disease produces drastic economic losses worldwide. According to the International Committee on Taxonomy of Viruses, ND is caused by virulent strains of NDV, namely, Avian avulavirus 1, which was later changed to Avian orthoavula virus serotype 1 and classified into the new subfamily Avulavirinae of the family Paramyxoviridae [4]. Importantly, two surface glycoproteins are located in the NDV envelope; the hemagglutinin neuraminidase (HN) protein, which is responsible for attachment of the virus to the host cell, and the F protein which is essential for fusion of the virus to the host cell membrane [5]. The F and HN proteins represent the key goals of the immune response against NDV [6]. Depending on whole-genome sequence and F gene sequence, all NDV strains are classified into one serotype, which includes two classes of NDV: class 1 and 2. A single genotype was found in class 1 (1.I) which contains nonvirulent NDV strains that are usually asymptomatic in aquatic wild birds. On the other hand, the class 2 NDV strains are classified into 21 genotypes (2.I–2.XXI) [7].

In accordance with their distribution, NDV strains with genotypes II, VI, and VII are the main identified NDV genotypes in Egypt and other North African countries [8]. In this respect, NDV genotype VII was first identified in Egypt in 2011 [9]. NDV F gene sequence analysis is usually used for NDV virulence determination as it is the main determining factor of virus virulence according to which the NDV isolates are categorized into different genotypes [10,11,12,13]. In addition, the amino-acid sequence of the NDV F0 glycoprotein precursor cleavage site is considered the main site for major virus virulence changes [13,14]. NDV virulence classification is usually measured by the intracerebral pathogenicity index (ICPI) in 1 day old chicks, using a scale of 0–2. High scores close to 2 are considered very virulent strains [1,15]. The mean death time (MDT) can be used for determination of NDV virulence, as velogenic strains have an MDT of less than 60 h [10,11]. Velogenic NDV strains can be further subdivided into viscerotropic, which cause severe intestinal and visceral hemorrhages, and neurotropic, which cause severe neurologic clinical signs and encephalitis [2,16,17,18]. Among poultry, ND has a wide prevalence of infection in over 200 kinds of birds [2,3,19]. In many countries, novel genotype VII displayed marked virulence and wide tissue tropism. Moreover, it was heavily shed from oral and cloacal secretions with an elevated infection and replication potential, suggesting its quick spread across geographic zones [20].

It is worth mentioning that the pathogenicity of NDV depends on different factors including species, bread, age, maternal immunity, reproduction status, and the route of infection [21]. It seems that the routes of infection affected the severity of clinical signs observed in ducks infected with Newcastle disease via intramascualr (I/M), intranasal (I/N), and intraocular (I/O) routes, whereas ducks infected via the I/M route showed higher clinical signs and mortalities than birds infected via I/N and I/O routes. There are dozens of reports investigating the pathogenicity of ND in chicken. However, to the best of the authors’ knowledge, there are no detailed time-course comparative clinicopathological studies of NDV infection with different routes of inoculations including nasal, ocular, choanal, and mixed nasal and ocular routes in white Ross commercial chicken [21]. In the current study, a new isolated virulent field NDV strain was used to induce infection in white Ross commercial chickens via different inoculation routes. Pathogenesis, comparative clinicopathological features (5 and 10 days post inoculation), and the role of different routes of infection by NDV were investigated. Given the paucity of information on the disease from NDV genotype VII outbreaks, our study also compared the severity and distribution of lesions in different organs in chicken infected with different routes of inoculation in a time-course manner in an attempt to highlight the pathogenesis of the viral infections in chicken. To the best of our knowledge, there are no previous reports providing a detailed comparative pathogenesis of different inoculation routes of NDV in chicken.

## 2. Material and Methods

### 2.1. Ethical Considerations

The ethical approval of the study was obtained from the Research, Publication, and Ethics Committee of the Faculty of Veterinary Medicine, Kafrelsheikh University, Egypt. The ethical approval code number of the study is KFS-2019/08.

### 2.2. Study Area and Collection of Clinical Specimens

This study was conducted on 12 farms of 18–32 day old broiler chickens in Dakahlia Governorate, Egypt during the year 2019. Tracheal swabs were collected from diseased birds suspected to be infected with NDV (pool of five birds/farm). The swabs were collected on brain–heart infusion broth, pH 7.4, containing antibiotics (2000 IU/mL penicillin, 2 mg/mL streptomycin, 50 mg/mL gentamycin, and 1000 IU/mL fungizone) [22]. The swabs were then transported in an ice box directly to the National Laboratory for Veterinary Control on Poultry Production, Animal Health Research Institute, Cairo, Egypt for molecular identification of NDV, virus isolation, and titration.

### 2.3. Molecular Identification of NDV

#### 2.3.1. Viral RNA Extraction

Tracheal swabs were centrifuged at 2000 rpm for 15 min, and the supernatant fluids were used for viral RNA extraction with a commercial kit QIAamp^®^ Min Elut^®^ Virus Spin Kit (QIAGEN, Hilden, Germany) following the kit instructions. A positive control velogenic NDV strain from a previous study (Mans 1 strain; GenBank accession no. MN537832) was included in the RRT-PCR and RT-PCR [23]. The extracted RNA was then stored at −80 °C until use in RT-PCR.

#### 2.3.2. Real Time RT-PCR (RRT-PCR)

Real-time RT-PCR was used for screening of velogenic and mesogenic NDV strains in 12 collected tracheal swabs using a previously reported specific primer set for amplification of the 101 bp F gene fragment as described in Table 1. Real time RT-PCR was applied using the commercial kit QuantiTect Probe RT-PCR Master Mix (QIAGEN, Hilden, Germany) according to manufacturer guidance. RT-PCR was conducted in a Stratagene MX3005P real-time PCR System (Stratagene, California, CA, USA) which adjusted to one cycle of 50 °C for 30 min and another cycle of 94 °C for 15 min, followed by 40 cycles of three steps: 94 °C for 15 s, 52 °C for 30 s, and 72 °C for 10 s.

#### 2.3.3. RT-PCR

RRT-PCR-positive samples were used in RT-PCR for amplification of the 370 bp fragment of the virulent NDV F gene using QIAGEN OneStep RT-PCR Kits (QIAGEN, Hilden, Germany) in accordance with the kit instructions. RT-PCR was applied using a previously reported set of primers, shown in Table 1 [24]. The T3 Biometra thermal cycler (Biometra, Germany) was adjusted to a single cycle of 50 °C for 30 min and another cycle at 94 °C for 2 min, followed by 40 cycles of three steps (95 °C for 30 s, 50 °C for 45 s, and 72 °C for 1 min), with a final cycle of 72 °C for 10 min. PCR products were analyzed by 1.5% agarose gel electrophoresis, and then visualized with a UV transilluminator (Biometra, Germany).

**Table 1:** Details of oligonucleotide primers and probe used for partial amplification of NDV F gene in RRT-PCR and RT-PCR.

Primer	Sequence (5′–3′)	Product Size (bp)	Reference
F+4839	TCCGGAGGATACAAGGGTCT	101	[25]
F+4894	[FAM]AAGCGTTTCTGTCTCCTTCCTCCA[TAMRA]		
F−4939	AGCTGTTGCAACCCCAAG		
F 330	AGGAAGGAGACAAAAACGTTTTATAGG	370	[24]
R700	TCAGCTGAGTTAATGCAGGGGAGG		

#### 2.3.4. Sequencing and Phylogenetic Analysis

The RT-PCR product of the selected sample (sharpest band) was removed from the gel. QIAquick PCR gel purification kits (QIAGEN, Valencia, CA, USA) were used for purification of DNA from the gel according to manufacturer’s instruction. The purified RT-PCR product was sequenced in an Applied Biosystems^®^ 3130 Genetic Analyzer (Applied Biosystems, Foster City, CA, USA) using Big Dye Terminator v3.1 Cycle Sequencing Kit’s (Applied Biosystems, Foster City, CA, USA). The obtained nucleotide sequences were deposited in GenBank (http://www.ncbi.nlm.nih.gov/Genbank, accessed on 13 August 2020) with accession number MT887290 and then analyzed by ClustalW2 (https://www.ebi.ac.uk/Tools/msa/clustalw2/, accessed on 13 August 2020). The ClustalW2 output files were aligned with other sequences from the GenBank database using MEGA X software (neighbor joining and maximum likelihood phylogenic trees constructed with 1000 repeat bootstrap tests) and the ClustalW algorithm of BioEdit software Version 7.1 [26,27].

### 2.4. Propagation of NDV in Embryonated Chicken Eggs

Ten day old specific pathogen-free (SPF) embryonated chicken eggs (ECEs) were obtained from Nile SPF farm, Fayoum, Egypt and used in NDV propagation for three successive passages: titration of NDV, MDT, and ICPI. The supernatant (0.2 mL) of the NDV-CH-EGYPT-F42-DAKAHLIA-2019 strain and a negative control sample from healthy chickens were inoculated into the allantoic sac of five SPF ECEs/sample according to standard inoculation procedures described elsewhere [28]. The inoculated ECEs were incubated at 37 °C with daily candling; any deaths during the first 24 h post inoculation (pi) were discarded. ECEs with dead embryos 48–96 h pi were examined for embryonic lesions, and their allantoic fluids were harvested. Any ECEs with live embryos 96 h pi were chilled at 4 °C for 12 h, and then their allantoic fluids were harvested. Collected allantoic fluids of the third egg passage were stored at −80 °C until further use.

### 2.5. Pathogenicity Testing of NDV-CH-EGYPT-F42-DAKAHLIA-2019 Isolate

#### 2.5.1. Mean Death Time (MDT)

Tenfold serial dilution (10^−6^ to 10^−9^) of fresh infective allantoic fluid was prepared in a sterile phosphate-buffered saline (PBS) from the strain “NDV-CH-EGYPT-F42-DAKAHLIA-2019” as described elsewhere [29]. About 0.1 mL from each dilution was inoculated into the allantoic sac of five SPF ECEs (10 days old). The inoculated eggs were incubated at 37 °C and examined twice daily for 7 days; the death time of each embryo was recorded, the MDT was calculated, and the pathotype was determined as described elsewhere [10,11].

#### 2.5.2. Intracerebral Pathogenicity Index (ICPI)

In this step, SPF ECEs (*n* = 20) were incubated at 37 °C until hatching, and then used for determination of the NDV-CH-EGYPT-F42-DAKAHLIA-2019 strain ICPI [30]. NDV titer in the freshly harvested allantoic fluid was firstly determined by hemagglutination inhibition test (HI) as previously described by OIE (2019) [30]. The HA titer of NDV-CH-EGYPT-F42-DAKAHLIA-2019 isolate allantoic fluid was 2^6^; this allantoic fluid was then diluted tenfold with sterile antibiotic-free PBS and used in ICPI determination. One day old chicks (24 h after hatching) were inoculated intracerebrally with 0.05 mL of diluted virus into each of 10 chicks, whereas the other 10 chicks were inoculated intracerebrally with 0.5 mL of PBS (negative control). Topical anesthetic cream was applied at the inoculation site and then left for 2 min before virus inoculation. The chicks were examined daily for 8 days post inoculation (pi). At each observation, each bird was scored as follows: 0 = normal, 1 = sick, 2 = dead; the ICPI was then calculated as the mean score/bird/observation (total score of the 10 birds at 8 days divided by 80) as described by OIE (2019) [30]. Velogenic NDV is indicated by a high ICPI close to the maximum score of 2.0, while lentogenic virus is indicated by an ICPI close to the minimum score of 0.0.

### 2.6. Comparative Assessment of Velogenic NDV (Sub-Genotype VII.1.1) Pathogenicity in Chickens Using Different Inoculation Routes

#### 2.6.1. Experimental Chicks

One day old white Ross male commercial chicks (*N* = 100) were obtained from a commercial hatchery and raised in insulated pens with all essential biosecurity precautions to avoid cross-infection between experimental groups. Birds were acclimated for 27 days before the beginning of the experiment. Food and water were freely available to the birds throughout the experimental period without the application of medicinal additives or vaccines. Chicks were raised according to all relevant Egyptian legislations and the international animal welfare guidelines.

#### 2.6.2. NDV Strains

The NDV-CH-EGYPT-F42-DAKAHLIA-2019 strain was used for experimental infection of susceptible chicks. This strain was identified in this study as sub-genotype VII.1.1 and proven to be a velogenic strain by MDT and ICPI.

#### 2.6.3. Virus Titration

NDV-CH-EGYPT-F42-DAKAHLIA-2019 strains identified in the present study were titrated in SPF ECEs. Serial dilutions (10^−1^–10^−9^) were prepared from the allantoic fluid collected from the third egg passage of virus propagation, and 200 µL from each dilution was inoculated in 10 day old ECEs via the allantoic route (five ECEs per dilution). Five days pi, the embryo infective dose 50 (EID_50_) was calculated, according to Reed and Muench (1938) [31]. The virus titer of the tested isolate was 10^7^ EID50/mL

#### 2.6.4. Experimental Design

**Figure 1:**
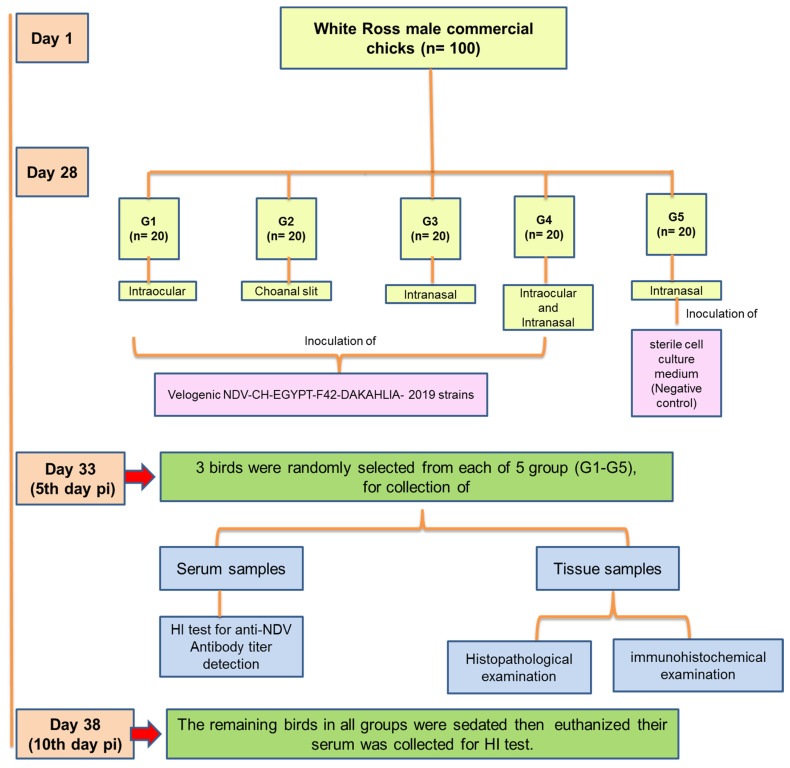
Diagram of the experimental design for the comparative assessment of velogenic NDV pathogenicity in chickens using different inoculation routes.

The experimental design of the comparative assessment of velogenic NDV in chickens using different inoculation routes is displayed in Figure 1. Experimental chicks (*n* = 100) were randomly divided at 28 days of age into five groups (G1–G5). Each group was composed of 20 birds each, inoculated with NDV-CH-EGYPT-F42-DAKAHLIA-2019 strains via a different route of inoculation. The experimental groups were physically separated from each other with strict biosecurity measures to prevent cross-infection between groups. Birds in G1–G4 were inoculated with 0.1 mL of NDV-CH-EGYPT-F42-DAKAHLIA-2019 (10^6^ EID_50_/bird), while G5 was an uninfected control group in which the birds were intranasally inoculated with a sham inoculum of sterile cell culture medium. G1, G2, and G3 birds were inoculated via the intraocular, choanal slit, and intranasal routes, respectively, whereas G4 birds were inoculated via both intranasal and intraocular routes. After NDV inoculation, each group was raised in physically separated pens and examined twice daily for clinical signs, morbidity, and mortality rates. On the fifth day pi, three birds from each group were randomly selected and injected intramuscularly with diazepam tranquilizer (2.5 mg/kg body weight) for sedation. Blood samples were collected from the wing vein for hemagglutination inhibition test (HI); then, the birds were slaughtered humanely, and their organs (brain, eyelid, nostrils, trachea, lung, proventriculus, gizzard, intestine, heart, liver, pancreas, bursa of Fabricius, thymus, cecal tonsils, and spleen) were collected in 10% buffered formalin for histopathological and immunohistochemical examination [32]. Later on, by the 10th day pi, the remaining birds were sedated and euthanized, and their blood and organs were collected and examined as previously described.

#### 2.6.5. Histopathological Examination

As previously mentioned, tissue samples were collected on the fifth and 10th day pi from different organs (brain, eyelid, nostrils, trachea, lung, proventriculus, gizzard, intestine, heart, liver, pancreas, bursa of Fabricius, thymus, cecal tonsils, and spleen) and fixed in 10% neutral buffered formalin. Samples were then processed using the paraffin embedding technique. Sections were cut at 4–5 μm thickness, stained with hematoxylin and eosin (H&E), and examined by light microscope for any histological alteration, as described elsewhere [33].

#### 2.6.6. Immunohistochemical Examination (IHC)

In this step, tissue sections were applied on poly-l-lysine-coated slides, which were deparaffinized and rehydrated as a routine tissue technique. Heat-induced antigen retrieval, blocking of nonspecific protein binding, and endogenous peroxide application were performed. Tissue sections were then incubated with a polyclonal primary antibody (rabbit anti-NDV Ig) overnight, which was kindly provided by the Faculty of Veterinary Medicine, Cairo University, Cairo, Egypt. This step was followed by incubation with horseradish peroxidase-conjugated goat polyclonal secondary antibody to rabbit Ig (SM802 EnVision^TM^ FLEX/HRP). Color was developed using 3,3’-diaminobenzidine (DAB) substrate (DM827 EnVision^TM^ FLEX DAB+ Chromogen) and counterstained with Mayer’s hematoxylin, before being observed under an optical microscope at 200× magnification [34]. Control negative samples were established by adding saline instead of the primary antibody and used as a reaction guide. To assess the specificity of the antibody and the extent of nonspecific labeling, rabbit IgG, polyclonal-Isotype Control (ab37415) (abcam, Cambridge, UK) was used instead of the primary antibody (isotype control) to optimize the technique and assess the presence of nonspecific staining.

#### 2.6.7. Anti-NDV Antibody Titer Detection

The hemagglutination inhibition test was used for detection of anti-NDV antibody titer. Briefly, blood samples were collected from sedated birds on the fifth and 10th day pi in plain tubes from all experimental groups. The blood was left to clot and then centrifuged at 3000 rpm for 10 min for serum separation. Serum was then collected and stored at −20 °C until use in the HI test according to OIE (2012) [11].

#### 2.6.8. Statistical Analysis

The collected data were coded, processed, and analyzed using the Statistical Package for Social Sciences (SPSS) version 15 for Windows^®^ (SPSS Inc, Chicago, IL, USA). The geometric mean of NDV (log base 2) and of HI antibody titers (±standard deviation) was quantitatively presented in all groups. A paired *t*-test was used for comparison within groups. Student’s *t*-test was used to compare between two groups. The F-test (one-way ANOVA) was also used to compare more than two groups. A *p*-value <0.05 was considered statistically significant.

## 3. Results

### 3.1. Clinical Signs and Postmortem Lesions in Naturally Diseased Birds

As mentioned above, tracheal swabs were collected from diseased birds (18–32 days old) suspected to be infected with NDV. These birds were exhibiting nervous symptoms, respiratory difficulties, and high mortality (70–85%). Furthermore, tracheitis, pneumonia, enlarged hemorrhagic spleen, hemorrhagic edematous cecal tonsils, pinpoint hemorrhages on the tips of the periventricular gland, and hemorrhagic enteritis were observed during postmortem examination.

### 3.2. Molecular Identification of NDV

#### 3.2.1. Real-Time RT-PCR

The present study revealed that 10 out of 12 tested samples (83.3%) were positive according to RRT-PCR with cycle threshold (Ct) values ranging from 17.33 to 32.20.

#### 3.2.2. RT-PCR

RT-PCR was used for partial amplification of the NDV F gene (370 bp) from 10 RRT-PCR-positive samples. In this respect, eight samples (80%) out of 10 tested samples had an amplicon size of 370 bp.

#### 3.2.3. Sequencing and Phylogenetic Analysis

**Figure 2:**
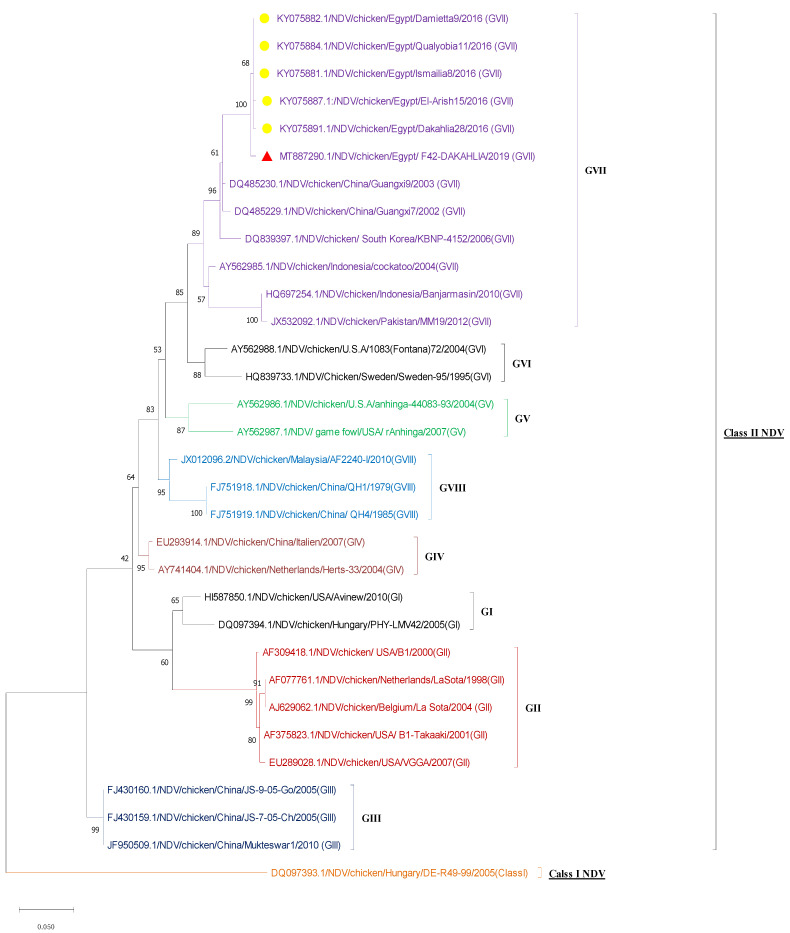
Neighbor joining phylogenic tree of NDV F gene partial sequences from different genotypes with 1000 bootstrap tests. Our strain (red triangle) was aligned with other Egyptian GVII strains (yellow dots) obtained from GenBank.

**Figure 3:**
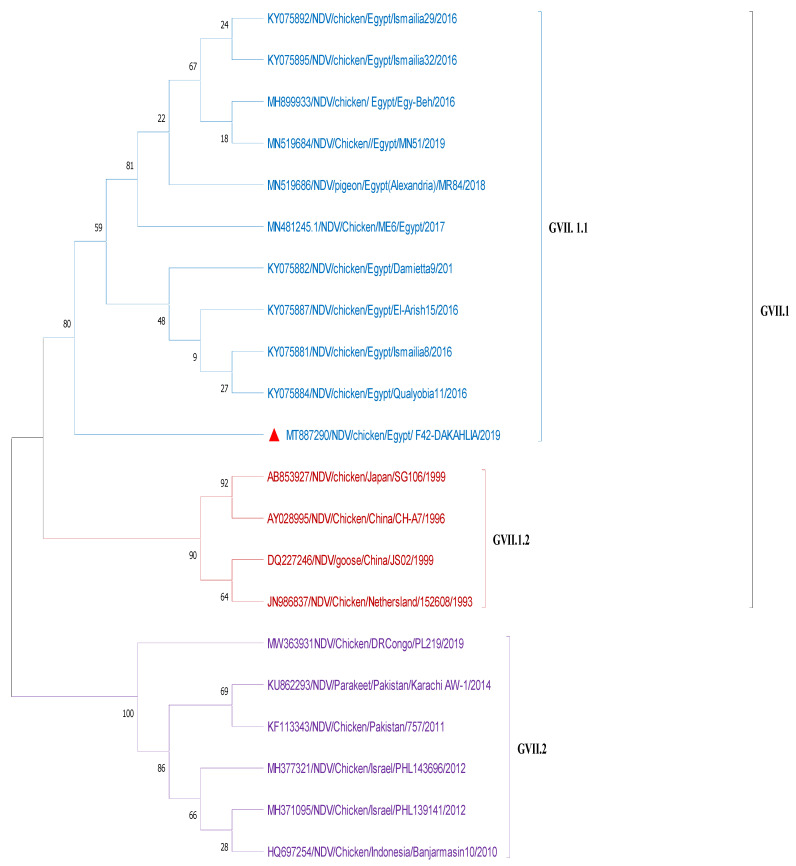
Maximum likelihood phylogenic tree of NDV F gene partial sequences of GVII sub-genotypes with 1000 bootstrap tests. Our strain (red triangle) was aligned with other Egyptian GVII.1.1 strains obtained from GenBank.

**Figure 4:**
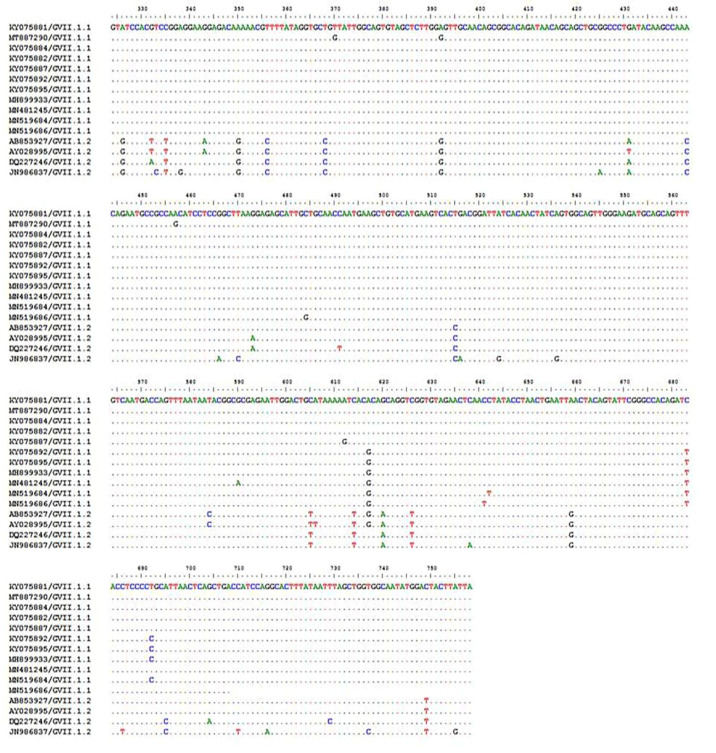
Nucleotide sequence alignment of the F gene of NDV GVII.1 strains using KY075882/Egypt/Damietta9 as a reference strain; similarities are presented as dots, while differences are presented as letters.

**Figure 5:**
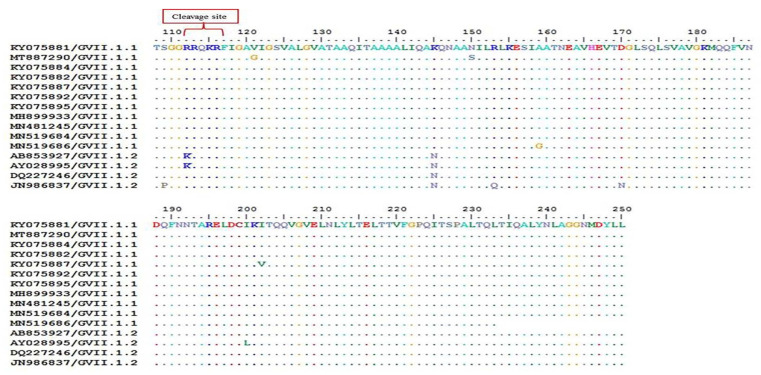
Deduced amino-acid sequence alignment of the F gene of NDV GVII.1 strains using KY075882/Egypt/Damietta9 as a reference strain; similarities are presented as dots, while differences are presented as letters.

The nucleotide and deduced amino-acid sequences of the NDV-CH-EGYPT-F42-DAKAHLIA-2019 strain F protein were aligned with other reference NDV strains from GenBank. As shown in Figure 2 and Table 2, the phylogenic neighbor joining tree possessing the two NDV classes (class 1 and 2) revealed that our strain was aligned with other Class 2 genotype VII NDV strains from Egypt with 99.3% nucleotide sequence identity (Damietta9, Qualyobia11, and Ismailia8 strains) and 99.07% nucleotide sequence identity (El-Arish15 and Dakahlia28 strains). Our strain was also closely related to other class 2 genotype VII NDV strains from other countries including China (Guangxi9 and Guangxi7 strains with 96.68% and 96.19% nucleotide sequence identity, respectively), South Korea (KBNP-4152 strain with 94.68%nucleotide sequence identity), Indonesia (Cockatoo and Banjarmasin strains with 94.43% and 90.35% nucleotide sequence identity, respectively), and Pakistan (MM19 strain with 90.07% nucleotide sequence identity). The phylogenic maximum likelihood tree possessing the sub-genotypes of NDV GVII (Figure 3) revealed that our strain belonged to sub-genotype GVII.1.1 as it was aligned with other Egyptian GVII.1.1 strains. Nucleotide sequence alignment of our strain with other GVII.1 strains using Bioedit software (ClustalW multiple alignment test) revealed that our strain had the same nucleotide sequence as other GVII.1.1 strains with two nucleotide substitutions (T370G and A392G), leading to amino-acid substitution at two sites (V121Gand N150S). Furthermore, as depicted in Figure 4 and Figure 5, the GVII.1.2 strains possessed several nucleotide and amino-acid substitutions in comparison to GVII.1.1 strains. Deduced amino-acid sequence analysis with Bioedit software (ClustalW multiple alignment test) confirmed that our strain is a velogenic strain according to the F protein amino-acid motif at the cleavage site (^112^ R–R–Q–K–R–F^117^).

### 3.3. Propagation of NDV in Embryonated Chicken Eggs

Ten day old SPF ECEs were used in NDV-CH-EGYPT-F42-DAKAHLIA-2019 strain propagation for three successive passages. The embryonic deaths were recorded at 48 h pi. Inoculated embryos were congested with subcutaneous hemorrhages all over the body and exhibited retarded growth and abnormal feathering.

### 3.4. Pathogenicity Testing of NDV-CH-EGYPT-F42-DAKAHLIA-2019 Isolate

The NDV-CH-EGYPT-F42-DAKAHLIA-2019 strain was classified as a velogenic strain according to the MDT (48 h) and the ICPI (1.83).

### 3.5. Comparative Assessment of Velogenic NDV Pathogenicity in Chickens Using Different Inoculation Routes

#### 3.5.1. Clinical Signs of Experimentally Infected Chickens

In the present work, clinical signs were first developed on the second day pi including a decrease in appetite and depression of NDV-infected birds. By the third day pi, the birds of G1–G4 appeared sleepy with a dropped and deviated head, along with reluctance to move and ruffled feathers, as well as abnormal respiratory sounds (hoarse chirps). Closed eyes with inflamed lids and more obvious abnormal respiratory sounds were clearly seen, especially in G1 and G4. On the other hand, greenish soft diarrhea was clearly noticed in G2. By the fourth day pi, watery eyes, ataxia, labored breathing and coughing were mainly observed in G1 and G2. On the fifth to sixth day pi, the previous clinical signs were exaggerated with more prominent nervous and respiratory manifestations in G1–G4. Deviation of head and neck with ataxia, suboptimal response of perching reflex, and incoordination were clearly noticed on the birds of G1 and G4. In G3, the mucoid nasal discharge was more copious and filled the nasal and oral cavities of infected birds. The severity of respiratory signs increased mainly on the seventh day pi in G1–G4, including tracheal rales, with closed eyes and inflamed infraorbital sinuses in G1, G3, and G4 with more gasping in G3. From the 10th day pi, all the observed clinical signs decreased with the exception of slight depression in G1–G4, with only one bird in G1 showing prolonged nervous signs (torticollis). In G3, birds recorded the highest mortality rate among infected groups, and their clinical signs were more severe. On the other hand, birds in G5 were healthy with no mortality throughout the experimental period. All the observed clinical signs, number of deaths, and mortality rates in different inoculated routes are fully described in Table S1.

#### 3.5.2. Gross Lesions of Experimentally Infected Chickens

On the fifth day pi, the most commonly observed lesions were hemorrhagic proventricular tips, hemorrhages in gizzard, and slightly thickened air sacs with congested cerebral and cerebellar blood vessels. Moreover, atrophied lymphoid organs (bursa, spleen, and thymus), swollen gallbladder congestion, and petechial hemorrhages in the thymuses and duodenum with a congested lung were noted. Mucoid discharges were observed in the nostrils, especially in the birds of G3. On the 10th day pi, the birds of G1 showed myocardial and hepatic necrotic areas with hemorrhagic enteritis, severely atrophied cecal tonsils, and a thymus with clearly congested brain vessels. However, pericardial edema began to appear in the birds of G2 with no remarkable necrotic lesions. Increases in pulmonary congestion and mucopurulent nasal discharge were detected in the birds from G3 and G4 with a clear pericardium edema and congested coronary arteries in G4. No postmortem lesions were recorded in G5 birds. A summary of gross lesions in different inoculated chickens with the NDV *CH-EGYPT-F42-DAKAHLIA-2019* strain via the intraocular, choanal slit, intranasal, and mixed intranasal and intraocular routes is presented in Table S2.

#### 3.5.3. Histopathological Examination

**Figure 6:**
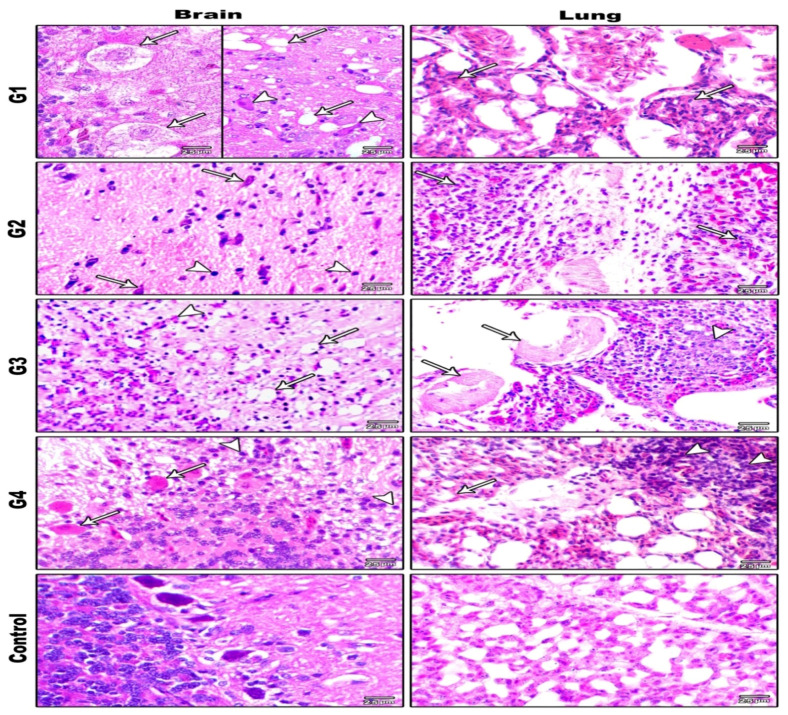
Histopathological findings of brain and lung in G1, G2, G3, and G4 challenged with NDV-VII (fifth day pi) and in the control group (H&E ×400). G1: Brain showing cerebellar Purkinje cell vacuolation (arrows) and cerebral spongiosis (arrows) associated with neuronal necrosis (arrowheads). G2: Brain showing cerebral neuronal necrosis (arrow) associated with gliosis (arrowheads). G3: Brain showing cerebral spongiosis (arrows) and micogliosis (arrowhead). G4: Brain showing cerebellar Purkinje cell necrosis (arrows) and micogliosis (arrowhead). G1: Lung showing hemorrhagic pneumonia associated with interstitial inflammatory cell infiltration and thickening of interalveolar septa (arrows) with distortion and narrowing of alveolar space. G2: Lung showing hemorrhagic pneumonia associated with interstitial inflammatory cell infiltration associated with collapsed alveoli (arrows). G3: Lung showing severe fibrinous pneumonia associated with fibrin deposition in parabronchi (arrows) and pulmonary consolidation with mononuclear cellular infiltration (arrowhead). G4: Lung showing pneumonia associated interstitial inflammatory cell infiltration and thickening of interalveolar septa (arrows), with distortion and narrowing of alveolar space and focal area of pulmonary consolidation (arrowhead). Control group brain and lung showing normal tissues with no obvious histopathological changes.

**Figure 7:**
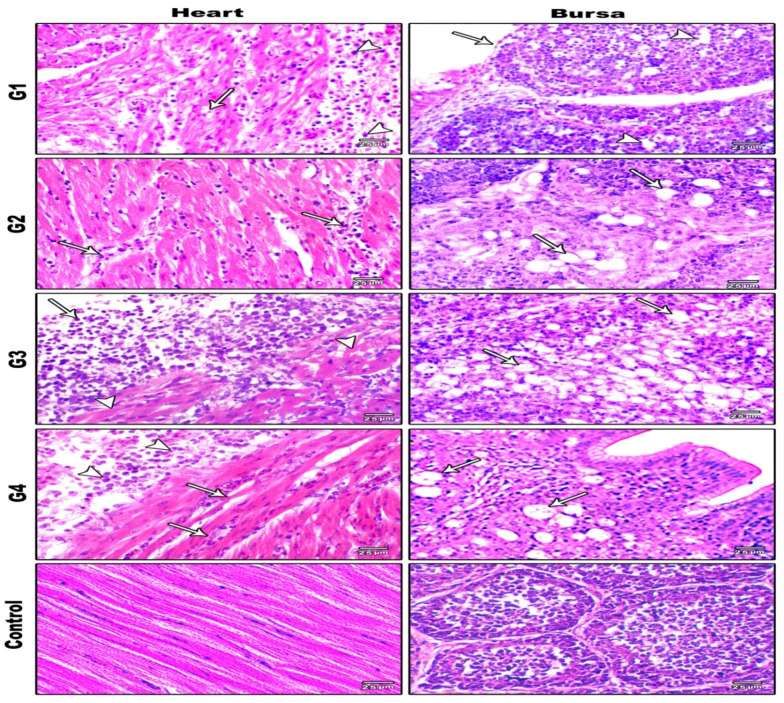
Histopathological findings of heart and bursa in G1, G2, G3, and G4 challenged with NDV-VII (fifth day pi) and in the control group (H&E ×400). G1: Heart showing pericarditis associated with oedema and inflammatory cell infiltration (arrowheads) and myocarditis associated with myocytes degeneration and necrosis (arrows). G2: Heart showing myocarditis associated with very mild myocyte degeneration and mononuclear cellular infiltration (arrows). G3: Heart showing pericarditis associated with mononuclear cellular infiltration (arrows) and fibrin deposition, along with myocarditis associated with diffuse myocyte necrosis (arrowheads). G4: Heart showing pericarditis associated with mononuclear cellular infiltration (arrowheads) and fibrin deposition, along with myocarditis associated with myocyte degeneration and mononuclear cellular infiltration (arrowheads). G1: Bursa showing epithelial lining necrosis (arrow) and lymphoid depletion (arrowheads). G2: Bursa showing lymphoid depletion (arrows). G3: Bursa showing severe lymphoid depletion (arrows). G4: Bursa showing severe lymphoid depletion (arrows). Control group heart and bursa showing normal tissues with no obvious histopathological changes.

**Figure 8:**
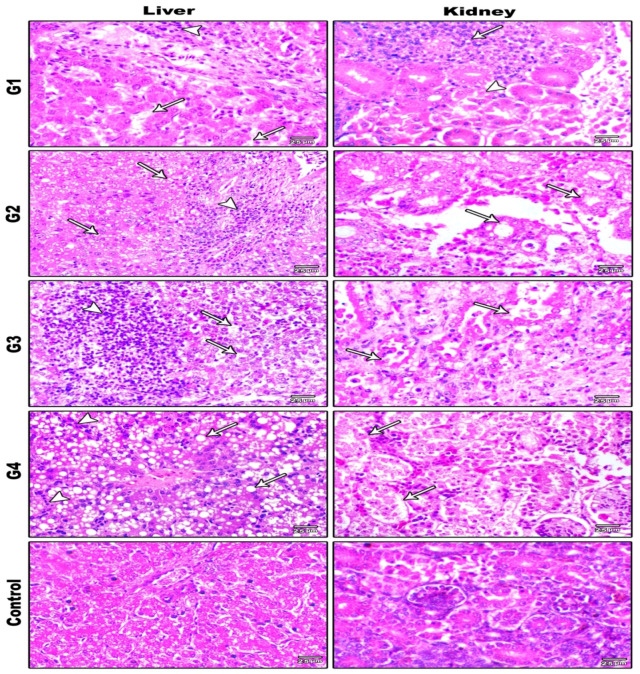
Histopathological findings of liver and kidney in G1, G2, G3, and G4 challenged with NDV-VII (fifth day pi) and in the control group (H&E ×400). G1: Liver showing hepatocyte degeneration (arrows) associated with interstitial mononuclear cellular infiltration (arrowhead). G2: Liver showing hepatocyte fatty change (arrows) associated with interstitial mononuclear cellular infiltration (arrowhead). G3: Liver showing hepatitis associated with hepatocyte necrosis (arrows) and mononuclear cellular infiltration (arrowhead). G4: Liver showing hepatitis associated with hepatocyte fatty change (arrows) and mononuclear cellular infiltration (arrowhead). G1: Kidney showing tubular necrosis (arrowheads) associated with interstitial mononuclear inflammatory cell infiltration (arrow). G2: Kidney showing tubular necrosis (arrow) associated with interstitial mononuclear inflammatory cell infiltration. G3: Kidney showing severe nephritis associated with diffuse tubular necrosis (arrows). G4: Kidney showing severe nephritis associated with glomerular cellular hyperplasia and tubular necrosis (arrows). Control group liver and kidney showing normal tissues with no obvious histopathological changes.

Histopathological observations of various examined organs within all challenged groups (G1, G2, G3, and G4) via different routes of inoculation are illustrated in Figure 6, Figure 7 and Figure 8. The severity and distribution of the lesions varied depending on the inoculation route, whereas the control group (G5) had no observed histopathological lesions. All histopathological findings are summarized in Table 3.

#### 3.5.4. Immunohistochemical Examination

Immune reactivity either in epithelial or inflammatory cells was observed in most of the examined organs. However, the intensity of immune reactive cells depended on the inoculated routes and was scored qualitatively as mild, moderate, and marked or severe immune reactivity. In G1 birds, there was mild to moderate immune reactivity in all examined organs, but marked immune labeling was observed in the epithelial cells of conjunctiva and cerebellar Purkinje cells. G2 birds developed a weak to mild immune reactivity in all organs except the conjunctiva, with the lowest expression in hepatocytes and cerebellar Purkinje cells.

**Figure 9:**
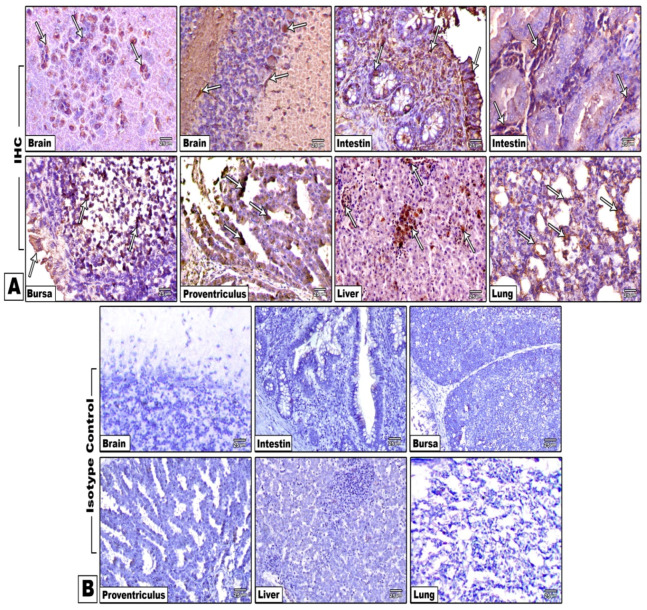
(**A**) Immunohistochemical examination of virulent NDV experimentally infected chicken tissues and immunolabeled with NDV antibody. There were immune reactive cells in neurons of the cerebrum, in Purkinje cells of the cerebellum, in epithelial cells, glandular epithelia, and inflammatory cells in the intestine, in epithelial cells and lymphoid cells of the bursa, in epithelial cells of the proventriculus, in hepatocytes and inflammatory cells, and in the alveolar epithelial lining of the lung (arrows). ABC method, counterstained with Mayer’s hematoxylin (×400). (**B**) Brain, intestine, bursa, proventriculus, liver, and lung were stained with a control isotype-matched antibody instead of the primary antibody to assess the specificity of the antibody and the extent of nonspecific labeling. ABC method, counterstained with Mayer’s hematoxylin (×400).

Surprisingly, a moderate to severe immune labeling was noticed in G3 birds, especially in the nasal epithelium, bronchial epithelial cells, lung, and hepatocytes, which showed intense reaction. In G4 birds, a moderate to severe immune reactivity was detected, especially in conjunctival and nasal epithelium, in addition to hepatocytes and Purkinje cells of the cerebellum, particularly on the 10th day pi. The immune reactivity of NDV for different inoculation routes in chicken is summarized in Figure 9A and Table 4. The stained organs with a control isotype-matched antibody instead of the primary antibody are shown in Figure 9B.

#### 3.5.5. Anti-NDV Antibody Titer Detection

The antibody titers against the NDV-CH-EGYPT-F42-DAKAHLIA-2019 strain are summarized in Table 5. There was an increase in antibody levels throughout the experimental period in all challenged groups as compared to the negative control birds with no significant difference in antibody titers among the challenged groups.

## 4. Discussion

Newcastle disease is a highly contagious viral disease of domestic and wild birds which causes drastic economic losses in poultry production. This disease is considered one of the major limiting factors facing the poultry industry worldwide, particularly in developing countries such as Egypt. Reviewing the available literature, several previous studies explored the pathogenesis of various NDV strains [17,35], as well as the host innate immunity and its relationship with the severity of the disease [36,37]. However, little is known about the effect of the type of infection route on the severity and the distribution of NDV lesions. The present study provides detailed findings highlighting the pathogenesis and comparative clinicopathological features in relation to the role of different routes of infection in birds experimentally infected with NDV in a time-course manner.

It is worth stating that molecular detection of the virus remains one of the most important steps in diagnosis of the virus [38]. Among others, RT-PCR amplification of the NDV F gene is considered the most common method for NDV detection with assessment of the NDV virulence [39]. Dimitrov et al. (2019) reported that NDV strains responsible for the fourth panzootic were aligned together into sub-genotype VII.1.1 [7].

As shown in our study, the phylogenic neighbor joining tree possessing the two NDV classes (class 1 and 2) revealed that our strain was aligned with other class 2 genotype VII NDV strains from Egypt with 99.3–99.07% identity. Meanwhile, the phylogenic maximum likelihood tree possessing the NDV GVII sub-genotypes revealed that our strain belonged to the sub-genotype GVII.1.1. These findings are in harmony with a previous study concluding that the sub-genotype VII.1.1 was the most predominant sub-genotype causing several NDV outbreaks in Egypt [9]. The cleavage site of velogenic NDV strains has multiple basic amino-acid residues between amino acids 113 and 116 (at least three arginines or lysines) at the C-terminus of the F2 protein with phenylalanine at the N terminus of the F1 protein (residue 117) [10,13]. In the current study, our isolate (NDV-CH-EGYPT-F42-DAKAHLIA-2019) had a velogenic NDV configuration of the cleavage site (four basic amino acids) (^112^ R–R–Q–R–R ^116^) at the C-terminus, as well as a phenylalanine (F) at residue 117. In addition to the characteristic velogenic cleavage site of our strain and its alignment with other velogenic sub-genotype VII.1.1 strains, this was also confirmed by the MDT (48 h) and the ICPI (1.83), which were in line with previous reports [2,11].

In the present work, field virus (NDV-CH-EGYPT-F42-DAKAHLIA-2019) was inoculated in the allontoic cavity of SPF ECEs for three passages. The isolated virus caused embryonic deaths at 48 h pi; inoculated embryos were congested with subcutaneous hemorrhages all over the body, along with retarded growth and abnormal feathering. These results are in accordance with some previous studies [23,40] showing that NDV causes embryonic death, along with blood-filled subcutaneous tissues of the head and prominent blood vessels covering the body. In contrast, another study reported that virulent strains of NDV might not kill the inoculated embryos [41]. For assessment of NDV-CH-EGYPT-F42-DAKAHLIA-2019 velogenic strains in 28 day old susceptible chickens via different inoculation routes, G1, G2, and G3 birds were inoculated via the intraocular, choanal slit, and intranasal routes, respectively, whereas G4 birds were inoculated via both intranasal and intraocular routes, and G5 birds were an uninfected control group. Importantly, the clinical signs, mortality pattern, and postmortem and histopathological lesions observed during the experimental period in different visceral organs, as well as the lymphoid organs and brain, indicated a highly virulent nature of the genotype VII pathotype of NDV, characteristic of velogenic ND virus infection in chickens [42,43,44,45,46,47]. In our study, the clinical manifestations associated with NDV-CH-EGYPT-F42-DAKAHLIA-2019 experimental infection via different routes of inoculation including anorexia, weight loss, depression, neck and leg abnormalities, paralysis, torticollis, watery greenish diarrhea, and hoarse chirps were similar to those reported elsewhere [1,17,48]. The clinical signs appeared in all challenged groups on the first day pi, represented by restlessness and decreased feed and water intake, indicating the high virulence of the isolated strain in the present study [49]. More severe illness was noticed on the third day pi in the birds inoculated via the intraocular and oculo-nasal routes as compared to the other experimental groups, in line with several previous reports [50,51].

In terms of mortality, two early deaths on the third day pi were recorded in birds of the mixed intraocular–intranasal inoculated group [52,53]. A similar early higher virus systemic distribution via the ocular route was reported in a recent study [54]. Although deaths began later on the fifth day pi in the other groups, these findings are in line with some previous studies [18,55]. In our present work, mortalities reached 30%, 40%, 45%, and 55% in the choanal inoculated group, ocular inoculated group, oculo-nasal inoculated group, and intranasal inoculated group, respectively. The recorded mortalities may be related to the higher systemic virus distribution, sever pancreatitis, and respiratory failure that occurred in the intranasal inoculated group. A mortality rate lower than 20% was observed in mesogenic strains in contrast to up to 100% mortality in velogenic strains [56]. Nervous signs were observed later on the fifth to seventh day pi, being more obvious in the intraocular and mixed intraocular–intranasal inoculated groups when compared with other groups. These findings may suggest the role of the ocular nerve in viral transmission to the brain. Similarly, nervous manifestations were reported in several previous studies [43,57]. Moreover, nervous signs consisting of torticollis and leg paralysis continued until the end of the experiment in the intraocular inoculated group, as explained by the severe cerebellar neuronal damage, suggesting caudal viral spread and neurotropism, which are common in velogenic neurotropic NDV [2,58]. Moreover, the virulent and nonvirulent NDV strains are able to simultaneously infect neurons, astrocytes, oligodendrocytes, and microglia cells in vitro [59] Greenish watery diarrhea was recorded in all challenged groups, but it was remarkable in the choanal inoculated group, which may suggest higher viral local replication through the GIT in this group compared to the remaining groups [56]. However, the respiratory signs, including abnormal respiratory sounds, nasal discharges, and labored respiration, were stronger in the intranasal inoculated and mixed oculo-nasal inoculated groups [46,47,56]. All inoculated groups showed nasal discharge, and the oral cavity was filled with mucous secretion upon necropsy, mainly in the intranasal and oculo-nasal inoculated groups due to intranasal virus inoculation, which caused copious mucus secretion in the nasal cavity along with stasis of GIT movement [43]. Furthermore, necropsy of challenged birds on the fifth day pi revealed severe petechial hemorrhages on glandular tips and on the mucosa of the proventriculus, as well as multiple foci of necrosis and hemorrhages in the small intestine [60]. The choanal slit inoculated group had less severe lesions in the digestive system than other groups. These findings may be due to the quick clearance and shedding of virus through diarrhea, as well as due to the protective role of the digestive tract associated with lymphoid aggregation, which may have led to the atrophied cecal tonsils in the choanal inoculated group. Another previous study reported similar symptoms including hemorrhagic cecal tonsils in the oculo-nasal, intraocular, and intranasal inoculated groups [61]. Furthermore, similar multiple white foci on the spleen were observed in all experimental groups [62], whereas, in the choanal challenged groups, the splenic foci were smaller in size than other groups. The observed lymphoid depletion, hemorrhagic enteritis, and hepatitis are common with virulent viscerotropic NDV [63].

On the other hand, the histopathological features of conjunctivitis, including edema, hemorrhage, and multifocal areas of epithelial necrosis associated with mononuclear inflammatory cell infiltrate, were more severe in the intraocular and mixed oculo-nasal inoculated groups, in line with a previous report [17]. Conjunctivitis was more severe on the inoculated side, and myositis was particularly induced in the mixed oculo-nasal inoculated group due to higher viral replication on the inoculated side [35,52,64,65]. Mild to severe rhinitis associated with hemorrhages and inflammatory cell infiltration and tracheitis associated with necrosis and ulceration of the epithelium were noticed in all experimental groups, in line with some previous studies [48,66]. Rhinitis and tracheitis were remarkable in the intranasal and mixed oculo-nasal inoculated groups. Moreover, interstitial pneumonia associated with edema, mononuclear inflammatory cell infiltration, and thickened interalveolar septa were detected in all challenged groups [48,62,67]. Fibrinous pneumonia was detected, mainly in the alveoli, in a few previous studies [48,62,67]. However, it was highly detected in our study in the alveoli and the bronchial lumen in the nasal and the mixed oculo-nasal inoculated groups and slightly detected in the choanal inoculated group, suggesting the high virulence of the current strain. Meanwhile, pneumonic features were severe in the intranasal inoculated and mixed oculo-nasal inoculated groups when compared with other groups. Although the bronchial lesions were more predominant on the fifth day pi in all experimental groups, except for the intraocular inoculated group, it was noticed on the 10th day pi. Interestingly, in the current study, the infected birds showed features of non-purulent encephalitis which is in accordance with some previous reports [48,51]. The encephalitic lesions varied in their severity among the inoculated groups. Severe lesions including encephalitis were seen in the oculo-nasal and intraocular inoculated groups when compared with other groups, which is in accordance with some previous reports. It should be stressed that the presence of NDV within the brain can cause vascular and neuronal damage, subsequently eliciting an inflammatory response such as perivascular cuffing, which can spread to surrounding astrocytes and microglia [68]. In the intraocular inoculated group, the neuronal necrosis of the cerebrum was less severe than in the cerebellum and medulla oblongata, which may suggest that caudal infection extended to the cerebral cortex. Pancreatic lesions included pancreatic acinar cell necrosis, and fatty changes in lymphocytes, macrophages, and few heterophils were recorded in all challenged groups, in agreement with several previous reports [51,66,69,70]. The intranasal inoculated group had the most necrotic pancreatic lesions in terms of severity and diffusion. Meanwhile, the fewest pancreatic lesions were seen in the choanal inoculated group when compared with other challenged groups. Cardiac lesions of the challenged groups revealed myocarditis features associated with edema, mononuclear cell infiltration, cardiac myocyte degeneration, and necrosis, as well as features of pericarditis, as also reported elsewhere [17,51,56]. In the current study, the cardiac lesions were more severe and remarkable in the intraocular, intranasal, and mixed oculo-nasal inoculated groups than the other groups. Severe cardiac lesions, mainly myocyte necrosis, were seen early in the intraocular, intranasal, and mixed oculo-nasal groups on the fifth day pi due to the high virulence of the virus strain, but not on the 10th day; myocarditis associated with myocyte degeneration and lymphocytic infiltration was not recorded in contrast to previous studies [17,51,71]. The myocyte necrosis was more diffuse in the intranasal and mixed oculo-nasal groups than the intraocular group, but it was not detected in the choanal group. Hepatic lesions including inflammatory cell infiltrations, hepatocytic hydropic degeneration, and fatty changes were noticed in all inoculated groups, although these findings were more remarkable in the intranasal group and associated with mild hepatocyte necrosis [51,53,56]. Meanwhile, IC inclusions in the hepatocytes were noticed in all groups except the choanal inoculated group, indicating higher viral systemic distribution for all routes of inoculation except the choanal route due to higher viral shedding, as mentioned previously.

In our study, kidney lesions were observed in renal tubules and glomeruli in all challenged groups except in the choanal group, where they mainly involved the renal tubules. The observed nephritis features were congestion, tubulo-interstitial nephritis, and glomerulitis. All challenged groups showed diffuse necrotic nephritis which involved the renal tubules [51,72,73,74], while the choanal group showed focal renal necrotic lesions, suggesting a wider systemic distribution of NDV through other routes of inoculation [62]. In terms of the lymphoid organs (bursa of Fabricius, spleen, thymus, and cecal tonsils), several lesions were noticed in all inoculated groups, including lymphocytic depletion and necrosis, in agreement with several previous reports [55,61,74,75]. The observed lymphoid depletion might be attributed to the multiplication of the virus and its presence in lymphocytes, which consequently greatly reduced the immune status of the birds [76]. The lymphoid organ lesions in the intranasal inoculated group were more severe than those in other groups and extended to the end of the experimental period.

Immunohistochemical staining is widely accepted as an accurate, quick, and economic technique for the identification of the virus and confirmation of the diagnosis of ND [69]. Importantly, ND viral antigens are mainly localized in the cytoplasm and nuclei of necrotic cells of the various organs, as well as in the epithelia, circulating monocytes, and alveolar macrophages in the lungs [77]. In the current study, NDV nucleoprotein immunolabeling revealed its wide systemic distribution, mainly in the lining epithelium of the conjunctiva, nostril, pulmonary alveoli, bronchial, kidney tubules, intestines, and bursa, in addition to hepatocytes, inflammatory cells (mainly macrophages and lymphocytes), and various neural cells and glia cells of the central nervous system. These findings are in line with some previous studies [20,49,77]. However, it should be borne in mind that the immunohistochemical expression of NDV nucleoprotein showed a variable degree of intensity among the examined organs in relation to the route of inoculation, along with more intense immunohistochemical expression of NDV nucleoprotein in inflammatory and epithelial cells recorded in the intranasal and mixed oculo-nasal inoculated groups. The lesions, as well as their incidence, nature, and affected organs, in relation to the infection route might yield insights for a better approach to vaccination. A previous study revealed that the immunization of chicks, through eye drop, induced the highest hemagglutination inhibition antibody titers at 20 days of age, followed by immunization through distilled water and through feed [54]. These findings revealed the efficacy of immunization through eye. Similarly, the intraocular route was found to be better than the intranasal and the drinking water routes [53].

## 5. Conclusions

Collectively, the present study concluded that experimental infection with the *NDV-CH-EGYPT-F42-DAKAHLIA-2019* strain via different routes of inoculation drove the systemic viral pantropism. Although the basic features of NDV infection were commonly observed in all birds infected with the *NDV-CH-EGYPT-F42-DAKAHLIA-2019* strain, there were several variations in relation to the route of viral inoculation. Among other routes, prominent respiratory manifestations and the highest mortality rate were recorded in the intranasal inoculated group which might be attributed to the high systemic distribution and respiratory failure. In addition, the lowest clinical manifestations and postmortem lesions were noticed in the choanal inoculated group as compared to other groups due to quick clearance and shedding of the virus during diarrhea, as well as due to the protective role of the digestive tract associated with lymphoid aggregation. Features of non-purulent encephalitis were clearly seen in the intraocular inoculated group, with signs of possible caudal viral spread, indicating the role of the optic nerve in viral pathogenesis and viral neuronal tropism. The route of infection should be considered when conducting a pathogenicity study on new isolated strains of NDV, especially when comparing their results with other studies. The present findings might be helpful for designing vaccination strategies against NDV. Further studies are needed to validate the influence of various vaccination routes on the virus distribution and infectivity.

## Figures and Tables

**Table 2:** Identity and diversity (%) between our strain (MT887290/Egypt/F42-DAKAHLIA) strain and other GVII.1.1 strains from GenBank.

	% Diversity													
			1	2	3	4	5	6	7	8	9	10	11	12
**% Identity**	1	MT887290/Egypt/F42-DAKAHLIA		99.3	99.3	99.3	99.07	99.07	96.68	96.19	94.68	94.43	90.35	90.07
	2	KY075882/Egypt/Damietta9	0.7		100	100	99.77	99.77	96.93	96.44	94.94	94.69	90.94	90.37
	3	KY075884/Egypt/Qualyobia11	0.7	0		100	99.77	99.77	96.93	96.44	94.94	94.69	90.94	90.37
	4	KY075881/Egypt/Ismailia8	0.7	0	0		99.77	99.77	96.93	96.44	94.94	94.69	90.94	90.37
	5	KY075887/Egypt/El-Arish15	0.93	0.23	0.23	0.23		99.54	96.68	96.19	94.69	94.44	90.67	90.09
	6	KY075891/Egypt/Dakahlia28	0.93	0.23	0.23	0.23	0.46		96.68	96.19	94.69	94.44	91.21	90.65
	7	DQ485230/China/Guangxi9	3.32	3.07	3.07	3.07	3.32	3.32		99.07	97.65	96.93	93.09	92.55
	8	DQ485229/China/Guangxi7	3.81	3.56	3.56	3.56	3.81	3.81	0.93		97.17	96.44	92.57	92.98
	9	DQ839397/South Korea/KBNP-4152	5.32	5.06	5.06	5.06	5.31	5.31	2.35	2.83		95.44	92.01	91.45
	10	AY562985/Indonesia/Cockatoo	5.57	5.31	5.31	5.31	5.56	5.56	3.07	3.56	4.56		94.65	94.12
	11	HQ697254/Indonesia/Banjarmasin	9.35	9.06	9.06	9.06	9.33	8.79	6.91	7.43	7.99	5.35		99.53
	12	JX532092/Pakistan/MM19	9.93	9.63	9.63	9.63	9.91	9.35	7.45	7.98	8.55	5.88	0.47	9.93

**Table 3:** Histopathological alterations in different organs in chickens inoculated with NDV-CH-EGYPT-F42-DAKAHLIA-2019 strain via the intraocular, choanal slit, intranasal, and mixed intranasal and intraocular routes.

		Conjunctival Route (G1)		Choanal Slit (G2)		Nasal Route (G3)		Mixed Conjunctival and Nasal Routes (G4)	
Days pi		5th Day pi	10th Day pi	5th Day pi	10th Day pi	5th Day pi	10th Day pi	5th Day pi	10th Day pi
Brain	Cerebrum	++ N.V	+ absent P.V, HEM, N.N, G	+ P.C, G, P.C, N.N	++	++ PC DEG, M, N.N, N.V	-	++ HEM meningo-encephalitis	+++ ventricle INFL
	Cerebellum	++/+++ HEM, P.L P.V, CON, N.N, SPN, stellatosis vasculitis	++ CON, G, P.L, M	+ P.L, CON, N.V, G, SPN	++ P.L, SPN	++ N.N P.L N.V	-	++ M, N.V, HEM meningo-encephalitis	+++
	Pones	++/+++	++ HEM, N.N, G	+ N.N, CON, N.V, G, meningitis	++ N.N, CON, G meningitis	+	-	++ meningo-encephalitis	+++
	Medulla	++/+++	++ HEM, M, N.N, G	+ N.N, CON, G, meningitis	++ N.N, CON, G meningitis	+	-	++ N.V, meningo-encephalitis	+++
Conjunctiva		++	+	−	−	−/+	−	+++	+ REG
Skin		−/+	+	−	−	−	−	+++	+
Nostril		++ N, INF, CON, EPITH Sloughing, Erosion	+ INF	++	++	+++ H.INFL, INF, I.B	+ REG	+++	++ maybe focal
Trachea		++ CON, ulcer	−/+ EPITH sloughing, INF	+/++ N, INF, CON, EDEMA	+/++ N, INF, CON, EDEMA	+++	+/++ REG	+++ FIB	+/++
Lung		++ INT PN/ HEM in ALV/INF in PA	+/++ INT PN, ALV EMPH/ INFL in (PA-BR)	+++ INT PN, Broncho- PN/ALV EMPH/(FIB, HEM) in (PA, ALV/INF inPA	+++ INT PN, Broncho- PN/ALV EMPH/(FIB, HEM) in (PA, ALV/INF inPA	+++ FIB Broncho-PN	+++ INT PN, Broncho- PN, lymphoid hyperplasia	+++ INT PN, Broncho- PN/VAS, HEM in (PA-BR)	+++ Broncho- PN/SeroFIB in (ALV-PA)
Gizzard		−/+ CON, focal N, EPITH sloughing	−/+ INF, CON, gland N	−/+	−/+	+ N. INFL	+	+ INF, HEM focal N, EPITH sloughing	−

Normal (−), mild (+), moderate (++), marked and severe (+++), alveoli (ALV), bronchi (BR), congestion (CON), degeneration (DEG), depletion (DEP), edema (EDE), emphysema (EMPH), epithelial (EPITH), fibrinous exudate (FIB), fatty vacuolation (F.V), gliosis (G), glomerular (GLOM), hemorrhage (HEM), hemorrhagic inflammation (H. INFL), hyper cellularity (HYPC), inclusion bodies (I.B), intra-cytoplasmic (I.C), infiltration (INF), interstitial (INT), inflammation (INFL), lymphocytic inflammation (L. INFL), malacia (M,) parabronchi (PA), perivascular cuffing (P.C), Purkinje cell loss (P.L), necrosis (N), necrotic inflammation (N. INFL), neural Vacuolation (N.V), pneumonia (PN), regeneration (REG), spongiosis (SPN), tubular (TUB), vacuolation (VAC), vasculitis (VAS).

**Table 4:** Immunolabeling for Newcastle disease viral nucleoprotein antigen by immunohistochemistry in chicken.

Organs	Conjunctival Route (G1)		Choanal Route (G2)		Nasal Route (G3)		Mixed Oculonasal (G4)	
	5	10	5	10	5	10	5	10
Conjunctiva	+++ E., F.	+++ E.	−	−	−	−	+++ E., F. cell	+++ E., F. cell
Nostril	+ M. (E., F.)	++ M., L.P.	++ M	++ M., L.P.	+++ M., S.M.	+++ M., L.P.	+++ M., L.P.	+++ M., L.P.
Lung	+ A.	++ A., P.B	++ A.	++ A., F.	+++ A., P.B	+++ A., P.B	++ A., P.B	++ A., P.B
Brain	++ P., G.	+++ P., G.,S	+ P., G.	+ P	++ P., G	++ P	++ P., G.	+++ P., G., S
Liver	+ F	++ F	+ F	+ F	++ F	+++ F	++ F	+++ F
Proventriculus	++	++ E.	++ E., F.	++	++	++ E., F.	++ E.	++
Intestine	++ E., F.	++	++ E. (V., GL.), F.	++ E., F.	++ E., (V., GL.)	++ E., F.	++ E., (V., GL.), F.	++ E.
Bursa	++	+	++	++	++	++	++	++

Negative reactivity (−); mild immunoreactivity (+); moderate (++); marked (+++), Purkinje cells (P.), epithelial cells (E.), inflammatory cells (F.), granule cells (G.), astroglia (S.), alveoli (A.), lamina propria (L.P.), mucosa (M.), submucosa (S.M.) villi (V.), gland (GL.).

**Table 5:** Log geometric mean of the hemagglutination inhibition (HI) antibody titer among the different experimental groups (*n* = 3).

Route/Days pi		5	10
G1	Intraocular	1.505 ± 0.301^a^	2.408 ± 0.521 ^a^
G2	choanal slit	1.605 ± 0.627 ^a^	2.308 ± 0.460 ^a^
G3	Intranasal	1.405 ± 0.460 ^a^	2.408 ±0.301 ^a^
G4	Mixed (I/N &I/O)	1.806 ± 0.301 ^a^	2.508 ± 0.348 ^a^
G5	Control	0.301 ± 0.0	0.1 ± 0.173

^a^ Significant differences (*p* < 0.05) between control and challenged chicken groups.

## Data Availability

The data that support the findings of this study are available on request from the corresponding author.

## Supplementary Materials

The following are available online at https://www.mdpi.com/article/10.3390/ani11123567/s1: Table S1. Summary of clinical signs and gross lesions in chickens inoculated with the CH-EGYPT-F42-DAKAHLIA-2019 strain via different routes; Table S2. Summary of gross lesions in chickens inoculated with the NDV CH-EGYPT-F42-DAKAHLIA-2019 strain via different routes.
