# Supplementary material for: Pathogenesis of Velogenic Genotype VII.1.1 Newcastle Disease Virus Isolated from Chicken in Egypt via Different Inoculation Routes: Molecular, Histopathological, and Immunohistochemical Study
Source: Animals (Basel). 2021 Dec 15;11(12):3567. doi: 10.3390/ani11123567 (PMC8698073; doi:10.3390/ani11123567)
Supplement: Supplementary file 1 [file animals-11-03567-s001.zip › animals-1462635-supplementary.pdf]

animals

Supporting Material

Pathogenesis of Velogenic Genotype VII.1.1 Newcastle Disease Virus isolated from Chicken in Egypt Via Different Inoculation Routes: Molecular, Histopathological and Immunohistochemical Study

Yassmin N. EL-Morshidy[1], Walied Abdo[2], Ehab Kotb Elmahallawy [*3], Ghada Allam Abd EL–Dayem[4], Ahmed El sawak[2], Nagwan El-Habashi[2], Samah M. Mosad [5], Maha S. Lokman [6,7], Ashraf Albrakati [8] and Samah Abou Asa[2]

[1] Department of Veterinary Pathology, Animal Health Research Institute (AHRI), Agriculture Research Center (ARC), P.O. Box 246 Dokki, 12618 – Giza, Egypt; Dr.jessy.mail@gmail.com

[2] Department of Veterinary Pathology, Faculty of Veterinary Medicine, Kafrelsheikh University, 33516, Kafrelsheikh, Egypt;waliedsobhy40@gmail.com (W.A); elsawak1953@yahoo.com (A.E); nagwan_hab@yahoo.com (N.E-H); ssabouasa@yahoo.com (S.A.A)

[3] Department of Zoonoses, Faculty of Veterinary Medicine, Sohag University, Sohag 82524, Egypt; eehaa@unileon.es

[4] Department of poultry diseases, Animal Health Research Institute (AHRI), Agriculture Research Center (ARC), P.O. Box 246 Dokki, 12618 – Giza, Egypt; anasesra@gmail.com

[5] Department of Virology, Faculty of Veterinary Medicine, Mansoura University, Mansoura 35516, Egypt; dr.sama786@yahoo.com

[6] Biology Department, College of Science and Humanities, Prince Sattam bin Abdul Aziz University, 11942, Alkharj, Saudi Arabia. 6; ms.hussein@psau.edu.sa

[7] Department of Zoology and Entomology, Faculty of Science, Helwan University, 11795 Cairo, Egypt. 7; ms.hussein@psau.edu.sa (M.S.L)

[8] Department of Human Anatomy, College of Medicine, Taif University, Taif 21944, P.O. Box 11099, Saudi Arabia; a.albrakati@tu.edu.sa

* Correspondence: eehaa@unileon.es (E.K.E)

animals

MDPI

Table S1. Summary of clinical signs and gross lesions in chicken inoculated with CH-EGYPT-F42-DAKAHLIA-2019 strain via different routes routes.

G		Intraocular route (G1)	Choanal slit route (G2)	Intranasal route (G3)	Mixed intranasal and intraocularroute (G4)
Day post infection (Dpi)/S	2-4Dpi/S	• Signs observed on 2 Dpi were slightly decreased food and water intake<br>• On 3-4 Dpi:<br>• Off food, ruffled feathers<br>• Closed eye , inflamed eyelid and watery eyes /+++ and Deviated head, +++<br>• lethargy and recumbence / and<br>• Greenish watery diarrhea /++<br>• Hoarse chirps (abnormal respiratory sound) /+	• Signs observed 2 Dpi were slightly decreased food and water intake<br>• On 3-4 Dpi:<br>• Off food, ruffled feathers<br>• watery eyes +<br>• lethargy and recumbence /++<br>• Deviated head/++<br>• Greenish watery diarrhea /+++<br>• Hoarse chirps /+++	• Signs observed on 2 Dpi were slightly decreased food and water intake<br>• On 3-4 Dpi:Off food, ruffled feathers<br>• Closed eye , inflamed eyelid and watery eyes /+<br>• Lethargy and recumbence /++<br>• Deviated head/+ and Hoarse chirps /+++<br>• Greenish watery diarrhea /++	• Signs observed 2 Dpi were slightly decreased food and water intake<br>• On 3-4 Dpi:<br>• Off food, ruffled feathers<br>• Closed and watery eyes /+++<br>• lethargy and recumbence /+++<br>• Deviated head/+++ and Hoarse chirps /++<br>• Greenish watery diarrhea /++<br>• 2 birds died 3rd Dpi and 1 bird died 4th Dpi
	5-7Dpi/S	• Signs progressed than 4th Dpi<br>• Nervous signs (deviated head and ataxia) /+++<br>• Mucoid nasal discharge ++<br>• Labored breathing /(+/++)<br>• 2 bird died on 5th Dpi,<br>• 4 birds died on 6th Dpi<br>• 2 bird died on 7th Dpi<br>• 3 birds were examined on 5th Dpi	• Signs as observed on 4th Dpi<br>• Mucoid nasal discharge /(++)<br>• Nervous signs/(++)<br>• Labored breathing /+++<br>• 3 bird died on 5th Dpi<br>• 1 bird died on 6th Dpi<br>• 2 bird died on 7th Dpi<br>• 3birds were examined 5th Dpi	• Signs severely progressed 4th Dpi<br>• Mucoid nasal discharge /(+++)<br>• nervous signs/+<br>• Labored breathing, grasping(+++)<br>• 3 birds died on 5th Dpi<br>• 4 birds died on 6th Dpi<br>• 3 birds died on 7th Dpi<br>• 3 birds were examined 5th Dpi	• Signs progressed than 4th Dpi<br>• Mucoid nasal discharge /(++/+++)<br>• nervous signs/ +++<br>• labored breathing /+++<br>• 3 birds died on 6th Dpi<br>• 3 bird died on 7th Dpi<br>• 3 birds were examined 5th Dpi
	8 up to 10Dpi/S	• As signs observed on 7th Dpi<br>• No mortalities<br>• 3 birds were examined on 10th Dpi	• Same signs observed 7th Dpi<br>• No mortalities<br>• 3 birds were examined 10th Dpi	• As signs observed on 7th Dpi<br>• 1 bird died on 10th Dpi<br>• 3 birds were examined 10th Dpi	• As signs observed on 7th Dpi<br>• 3 birds were examined on 10th Dpi
Mortality		40%	30%	55%	45%

animals MDPI

Table S2. Summary of gross lesions in chicken inoculated with NDV CH-EGYPT-F42-DAKAHLIA-2019 strain via different routes.

G	Intraocular route (G1)	Choanal slit route (G2)	Intranasal route (G3)	Mixed intranasal and intraocular (G4)
Gross lesions	• Mucoid discharges in nostrils &oral cavity/+<br>• Hemorrhage w congestion in proventriculus, gizzard and intestine / +++<br>• Congestion and hemorrhages of liver (5th Dpi)/++to+++<br>• brain congestion (5th Dpi)/+++<br>• fibrinous pericarditis, thickened air sac (5Dpi)/+<br>• Congested or hemorrhagic kidney (5th DPI)/++<br>• Hemorrhage w congestion in thymus (5th Dp)i/ ++<br>• watery eyes with inflamed eye lid<br>• atrophied hemorrhagic cecal tonsils (5th Dpi)<br>• Hemorrhage w congestion in lung (7th Dpi) / ++to+++<br>• atrophied spleen or w few white spots foci in spleen, 5th and 7^{th}Dp i<br>• Hemorrhage w congestion in lung/ 7th Dpi +++<br>• Hemorrhages myocardial5th Dpi/++<br>• necrotic areas on liver tips(10Dpi)	• Mucoid discharges in nostrils &oral cavity/++<br>• Hemorrhage w congestion in proventriculus, gizzard and intestine / - to+<br>• fibrinous pericarditis(5Dpi), thickened air sac /+ to++<br>• Hemorrhage w congestion in lung5^{th} Dpi / +to++<br>• Hemorrhage w congestion in thymus 5th Dpi /+<br>• atrophied cecal tonsils may w small hemorrhages (5th Dpi)<br>• Congestion and hemorrhages of liver (7th Dpi)/++<br>• brain congestion (5th Dpi)/+to++<br>• one white spots foci in spleen5^{th} and atrophied spleen 7th Dpi<br>• Congested or hemorrhagic kidney (7Dpi), friable pale kidney(10Dpi)	• Mucoid discharges in nostrils &oral cavity/+++<br>• Hemorrhage w congestion in proventriculus, gizzard and intestine/ +++<br>• Necrotic areas in small intestine (5th Dpi)<br>• Congestion and hemorrhages of liver (5th Dpi) /+++<br>• brain congestion (5th Dpi)/(- to+)<br>• fibrinous pericarditis(5Dpi) , thickened air sac /+++<br>• Hemorrhage w congestion in lung(5th Dpi) / +to++<br>• Congested or hemorrhagic kidney / 5th Dpi ++to+++<br>• Hemorrhage w congestion in thymus (5th Dpi)/ ++<br>• Hemorrhage w congestion in lung (5th Dpi)/+++<br>• Congestion of liver , brain congestion / ++<br>• atrophied hemorrhagic cecal tonsils (5th Dpi)<br>• Hemorrhages myocardial (5th Dpi) /++ to+++<br>• atrophied spleen or w few white spots foci in spleen5^{th} Dpi-7th	• Mucoid discharges in nostrils &oral cavity/++to+++<br>• Hemorrhage w congestion in proventriculus, gizzard and intestine /++ to +++<br>• Hemorrhage or congestion of liver(5th Dpi) /+++<br>• brain congestion (5th Dpi)/ +++<br>• atrophied spleen or w few White spots on spleen 5th Dpi-7th<br>• watery eyes with inflamed eye lid<br>• fibrinous pericarditis(5th Dpi), thickened air sac /+++<br>• Congested or hemorrhagic kidney (5th Dpi) / + ++<br>• Hemorrhage w congestion in thymus (5th Dpi)/+++<br>• Hemorrhage w congestion in lung/ (5th Dpi)/ +++<br>• Enlarged pale kidney (7th Dpi)<br>• atrophied hemorrhagic cecal tonsils (5th Dpi)<br>• Hemorrhages on duodenum<br>• Hemorrhages on myocardium (5th Dpi)/+++<br>• Necrotic areas in small intestine (7th Dpi)

Dpi=day post infection; w= with; /S=Score or degree of lesions; (+= mild; ++= moderate; +++= severe
